# Three cases of bacteremia caused by Vibrio cholerae O1 in Blantyre, Malawi.

**DOI:** 10.3201/eid0706.010629

**Published:** 2001

**Authors:** M A Gordon, A L Walsh, S R Rogerson, K C Magomero, C E Machili, J E Corkill, C A Hart

**Affiliations:** Queen Elizabeth Central hospital, Blantyre, Malawi.

## Abstract

We report three fatal cases of bacteremia (two adults, one neonate) caused by Vibrio cholerae O1 (Ogawa), which occurred in the context of a community outbreak of cholera diarrhea in Blantyre, Malawi. Only four cases of invasive disease caused by V. cholerae O1 have previously been reported. We describe the clinical features associated with these rare cases and discuss their significance.


### Three Cases of Bacteremia Caused by *Vibrio cholerae* O1 in Blantyre, Malawi

**Melita A. Gordon,*‡ Amanda L. Walsh,† Sheryle R.K. Rogerson,* Kingsley C. Magomero,* Chipulwa E. Machili,* John E. Corkill,‡ and C. Anthony Hart‡**

*Queen Elizabeth Central Hospital, Blantyre, Malawi; †Wellcome Trust Research Laboratories, Blantyre, Malawi; and ‡Royal Liverpool University Hospital, University of Liverpool, Liverpool, United Kingdom

We report three fatal cases of bacteremia (two adults, one neonate) caused by *Vibrio cholerae* O1 (Ogawa), which occurred in the context of a community outbreak of cholera diarrhea in Blantyre, Malawi. Only four cases of invasive disease caused by *V. cholerae* O1 have previously been reported. We describe the clinical features associated with these rare cases and discuss their significance.

*Vibrio cholerae* O1 and O139, the causative agents of cholera, are morphologically and biochemically identical to the other non-O1 *V. cholerae*, but antigenically, epidemiologically, and clinically distinct. Non-O1 *V. cholerae* can cause small outbreaks of diarrheal illness related to contaminated seafood. There are, however, numerous case reports of bacteremia caused by non-O1 *V. cholerae* in persons with predisposing conditions, most commonly cirrhosis (1) but also nephrotic syndrome, diabetes, hematologic malignancy, gastrectomy, and AIDS/lymphoma (2).

*V. cholerae* O1 and O139, by contrast, cause epidemic diarrheal disease. *V. cholerae* O1, in particular, is reputed to be noninvasive. Only three cases of bacteremia and one case of meningitis caused by *V. cholerae* O1 have been reported, from Australia (3), southern Africa (4), Pakistan (5), and Mexico (6) (Table). We report a series of three cases of bacteremia caused by *V. cholerae* O1 from a single center in sub-Saharan Africa (Queen Elizabeth Central Hospital [QECH], Blantyre, Malawi), which occurred in the context of a community outbreak of cholera.

### Cholera Outbreak

The three cases of bacteremia occurred during and after a cholera outbreak in Blantyre, Malawi, during March 1998, in which 178 adults (ages 15 to 68 years), 64 children (aged 1 month to 14 years), and 2 neonates were admitted to QECH with cholera diarrhea. Case 1 (neonate) occurred during the outbreak in March, and Cases 2 and 3 (adults) were among the sporadic cases at QECH during the following 12 months.

The first cases in the outbreak were identified by stool culture; thereafter, stool cultures were systematically obtained for 1 in 10 of suspected cases, to monitor the outbreak. Median intravenous fluid requirement for adult cases was 11 L (range 2 to 36). A single dose of doxycycline was prescribed for all suspected cases. There were two adult and two pediatric deaths during the March outbreak (overall death rate 1.6%), including Case 1 with cholera bacteremia. The three deaths not described below were attributed to acute severe dehydration, and one was associated with second-trimester abortion. During March 1998, adult patients were admitted and nursed adjacent to the wards in a cholera tent, where blood cultures were not routinely performed. After March 1998, sporadic cases (including Cases 2 and 3) continued to come to QECH; these patients were admitted to the general medical wards of the hospital. Blood cultures were routinely obtained for patients with fever and shock; such patients were cared for in the diarrhea bay of the medical wards.

### Case Reports

### Case 1 (Neonate)

A male twin was born in QECH in March 1998, at 34 weeks' gestation, by spontaneous vaginal delivery; he was breastfed. He was well until day 2, when he became hypothermic, hypoglycemic, and peripherally cyanosed. He had no diarrhea. Blood culture was taken, treatment with penicillin and gentamicin was begun, and expressed breast milk was fed by nasogastric tube, but the child died 13 hours later. Blood culture grew *V. cholerae* O1 at 24 hours (cloudy bottle). A stool culture was not taken.

The second twin followed a similar clinical course and died on day 2. Blood culture was negative. The mother was a healthy 21-year-old, with no diarrheal disease. We were unable to recall her for stool culture.

### Case 2 (Adult)

A previously healthy 45-year-old woman was admitted to QECH in September 1998 with profuse, watery diarrhea. She was afebrile, dehydrated, and tachycardic with thready pulses, and was managed with 11 L of intravenous Ringer's lactate followed by oral rehydration therapy (ORT). Her diarrhea became bloody, blood culture was taken, and nalidixic acid was given empirically. Over 36 hours her diarrhea resolved, her clinical state improved, and she was able to move around, but she died suddenly on day 4 after an

Address for correspondence: C. Anthony Hart, Department of Medical Microbiology, University of Liverpool, Duncan Building, 8th floor, Daulby Street, Liverpool L69 3GA, United Kingdom; fax: 440-151-706-5805; e-mail: cahmm@liv.ac.uk

Table. All reported cases of invasive disease caused by *Vibrio cholerae* O1, in chronological order

| Age, sex | Susceptibility | Clinical features | Outcome | Ref |
|---|---|---|---|---|
| 6 years, female | Autoimmune disease, achlorhydria | Diarrhea, severe sepsis syndrome | Survived after intensive therapy | 3 |
| 6 days, male | Neonate | Diarrhea, afebrile, neutrophilia, uremia | Died | 4 |
| 8 months, female | None | Diarrhea, febrile, neutrophilia | Survived with rehydration and antibiotics | 5 |
| 6 years, female | Chemotherapy | Meningitis, blood culture negative | Died | 6 |
| 2 days, male | Neonate | No diarrhea | Died | TR |
| 45 years, female | None | Diarrhea transiently bloody, afebrile | Died | TR |
| 65 years, female | None | Diarrhea, neutrophilia, renal failure secondary to dehydration | Died of renal failure after 2-3 weeks | TR |

TR = this report; see text.

unwitnessed collapse. *V. cholerae* O1 was grown at 24 hours (cloudy bottle). Stool culture had not been taken.

### Case 3 (Adult)

A previously healthy 65-year-old woman initially visited an outlying rural health center in February 1999 with sudden onset of profuse watery diarrhea. She was treated with 35 L of intravenous fluid followed by ORT for 4 days. She was not given antibiotics, her diarrhea ceased, and she was discharged. The water supply in her village was a covered well, and there was one simultaneous case of cholera diarrhea in the area, in a young woman, who fully recovered.

Over the next 3 days, Patient 3 had anuria, confusion, and shivering but no further diarrhea. She was taken to QECH, and on admission was afebrile, in shock, dehydrated, and confused. A clinical assessment of dehydration and sepsis prompted empiric management with intravenous rehydration, chloramphenicol, and gentamicin.

Blood tests revealed a leukocyte count of 22 x $10^9$/L (88% neutrophils), $Na^+$ 173 mmol/L (normal 135-145), $K^+$ 3.8 mmol/L (normal 3.5-5.0), and urea 71 mg/dL (normal 8-25). Liver function tests, urine examination, and chest X ray were normal. *V. cholerae* O1 was grown from blood at 36-48 hours (routine subculture) and was found to be sensitive to erythromycin but resistant to ampicillin, chloramphenicol, cotrimoxazole, and tetracycline; antibiotic therapy was changed accordingly. Blood culture taken after 7 days of treatment with erythromycin was negative. Rectal swab and urine cultures were negative. HIV serologic testing was negative. Despite rehydration and good subsequent urine output, she remained in renal failure with presumed acute tubular necrosis secondary to inadequate rehydration during her original diarrheal illness. She died 14 days after admission.

### Genomic Analysis

For adults, 5 mL of venous blood was incubated in a single aerobic culture bottle of 50 mL brain heart infusion broth containing sodium polyanetholesulphonate (E&O Laboratories, United Kingdom) at 37°C in air. For neonates, 2 mL of blood was incubated in 20 mL of broth in the same manner. Routine blinded subcultures on sheep blood agar incubated in $CO_2$ were performed at 24 and 48 hours and at 7 days. Bottles appearing cloudy were examined by Gram stain and then subcultured onto appropriate media, dependent on Gram stain findings. Antibiotic susceptibility testing was performed by disk diffusion. The organisms were identified biochemically and serologically as *V. cholerae* O1 (Ogawa).

Blood culture isolates from Cases 1 and 3 were available for subsequent genomic analysis. 16S rRNA sequence analysis was performed by using universal oligonucleotide primers (7). The 1,500-bp product was extracted from the gel and sequenced on an ABI PRISM system (Applied Biosystems, Perkin Elmer Corp, Foster City, CA). The 16S sequence was submitted to GenBank-BLAST Search for analysis. Multiplex polymerase chain reaction (PCR) was used to determine the presence of important virulence factors, namely, cholera toxin (*ctx*), toxin-regulated pilus (*tcp*), and the global regulatory element *toxR*, as described (8). Plasmids were extracted from control (*Escherichia coli* 39R861, *E. coli* V517) and test bacteria (Plasmid mini kit, Quiagen Ltd., Germany) and separated by electrophoresis. Pulsed-field gel electrophoresis (PFGE) of chromosomal DNA following digestion with the restriction endonuclease *Spe1* was performed. Clonal relatedness of the cholera isolates was assessed according to the criteria of Tenover (9).

16S rRNA sequence analysis confirmed both isolates as *V. cholerae*. Multiplex PCR amplicons of the appropriate size were detected for *ctxA* (301 bp), *tcpA* (618 bp), and *toxR* (900 bp). No plasmids were detected from the test isolates (the upper limit of plasmid size detection was 160 kbp). The two isolates were indistinguishable by PFGE of macrorestricted chromosomal DNA.

### Conclusions

This is the first reported series of *V. cholerae* O1 bacteremic cases. Biochemical, serologic, and genomic analysis confirmed the identity of the organisms as *V. cholerae* O1 (Ogawa).

These isolates could have been contaminants, arising on the ward or in the laboratory. Several features, however, make this unlikely. Skin was disinfected before blood was taken from the antecubital fossa, and a pure growth without skin contaminants was obtained in all three cases after 24 to 48 hours. Cases 2 and 3 postdated the main cholera outbreak, so the patients were not in a cholera tent and samples were not taken in an epidemic situation. There were no other coincident cases of cholera on the ward at the time. Moreover, in Case 3 a rectal swab culture was negative at patient admission, and the blood culture sample was taken in the general medical admissions area before the patient was transferred to the diarrhea bay. The blood culture specimens were handled in a research laboratory, in a separate building from the government laboratory where all stool cultures were performed. The three isolates could not be linked to any single technician or ward nurse, nor were they clustered in time. Finally, the high case death rate compared with the 1.6% overall death rate suggests that the isolates were of clinical relevance. Previously reported cases also show a poor outcome (Table).

Why did we observe bacteremia? All the cases we describe had unusual features or complications. Case 1 had no diarrheal illness, Case 2 had transient bloody diarrhea, and Case 3 was in an elderly woman who had renal failure secondary to inadequate initial rehydration. Invasive *V.cholerae* O1 disease has been associated with autoimmune disease, achlorhydria, and chemotherapy in two of the four previously reported cases (3,6), but our adult patients did not have known longstanding immunosuppression. HIV disease is common in Blantyre and is associated with bacteremia caused by *Streptococcus pneumoniae* and nontyphoid salmonellae (10), but no reports link HIV with severe or invasive *V. cholerae* O1 infections. *V. cholerae* O1 was grown from the stool of 5 of 77 Guatemalan AIDS patients; none had a fatal outcome, and 4 had only mild diarrhea. Three of these cases had enteric coinfection with *Cryptosporidium* or nontyphoid *Salmonella* (11).

Case 2 had transient bloody diarrhea, unlikely to be caused by *V. cholerae* alone. It is noteworthy that *V. cholerae* (unknown serogroup) and *Salmonella enterica* serotype Typhi were simultaneously isolated from blood in a 1932 case (12). Enteric bacterial coinfection may have facilitated mucosal invasion by *V. cholerae* in both these cases.

Cholera is well described in children <2 years of age, and breast feeding is protective (13). Cholera diarrhea is, however, extremely rare in neonates. (We found two cases with positive stool cultures during this outbreak.) Colostrum may offer potent protection among breastfed neonates in disease-endemic areas, mediated by specific immunoglobulin (Ig) A (14). Despite breastfeeding, however, Case 1 may have acquired *V. cholerae* O1 infection during birth from a mother with asymptomatic stool carriage (common during an outbreak). The early events of infection or invasion could have occurred before the first colostrum feed; the onset of symptoms on day 2 of life would be in keeping with this. The previously reported neonatal case (4) also had a healthy mother and onset of symptoms on day 5 of life.

The true incidence of bacteremia during this outbreak is unknown, as blood cultures were not routinely taken in the cholera tents. While *V. cholerae* O1 bacteremia is apparently a rare event, reported cases suggest that persons at risk include those with underlying immunosuppression (chemotherapy, autoimmune disease, achlorhydria), the elderly, and neonates. Enteric bacterial coinfection may play a role in invasion. There is no evidence that HIV infection is a risk factor. Intravenous rehydration and ORT remain the mainstays of successful treatment, but our experience reemphasizes the importance of antibiotics as adjunctive treatment.

What could be the route of invasion of *V. cholerae* O1? Intestinal M cells are enterocytes adapted to sample enteric organisms, which are then translocated to gut lymphoid tissue, where a specific sIgA response is generated. Viable *V.cholerae* O1 organisms are translocated across the mucosa in this manner by M cells. This has been proposed as the route by which *V. cholerae* O1 may in some circumstances cause bacteremic illness (15).

### Acknowledgments

The authors thank R.C. Read and S.B. Gordon for helpful comments on the manuscript, and M. Boeree, R. Broadhead, and the patients and staff of the Departments of Medicine and Pediatrics, College of Medicine, Malawi.

Dr. Gordon is a gastroenterologist, currently working as a Wellcome Trust Tropical Medicine Training Fellow in Blantyre, Malawi. Her research interests are the persistence of nontyphoidal salmonellae in HIV-infected adults following episodes of bacteremia.

### References

1. Ko WC, Chuang YC, Huang GC, Hsu SY. Infections due to non-O1 Vibrio cholerae in southern Taiwan: predominance in cirrhotic patients. Clin Infect Dis 1998;27:774-80.
2. Blanche P, Sicard D, Sevali GJ, Paul G, Fournier JM. Septicemia due to non-O1 Vibrio cholerae in a patient with AIDS [letter]. Clin Infect Dis 1994;19:813.
3. Rao A, Stoiber D. The Queensland cholera incident of 1977. The index case. Bull World Health Organ 1980;58:663-4.
4. Coovadia YM, Bhamjee A, Isaacson M. Vibrio cholerae bacteraemia in a newborn infant. A case report. S Afr Med J 1983;64:405-6.
5. Jamil B, Ahmed A, Sturm AW. Vibrio cholerae O1 septicaemia [letter] Lancet 1992;340:910-1.
6. Bustos EC, Gomez-Barreto D, Perez-Miravette A, Rodriguez RS. Vibrio cholerae O1 meningitis in an immunosuppressed child. Pediatr Infect Dis J 1996;15:772-3.
7. Edwards U, Rogall T, Blocker H, Emde M, Bottger EC. Isolation and direct complete nucleotide determination of entire genes. Characterisation of a gene coding for 16S ribosomal RNA. Nucleic Acids Res 1989;17:7843-53.
8. Mitra RK, Nandy RK, Ramamurthy T, Battacharya SK, Yamasaki S, Shimada T, et al. Molecular characterisation of rough variants of Vibrio cholerae isolated from hospitalised patients with diarrhoea. J Med Microbiol 2001;50:268-76.
9. Tenover FC, Arbeit RD, Goering RV, Mickelson PA, Murray BE, Persing DH, et al. Interpreting chromosomal DNA restriction patterns produced by pulsed field gel electrophoresis: criteria for bacterial strain typing. J Clin Microbiol 1995;33:2233-9.
10. Gordon MA, Walsh AL, Chaponda M, Soko D, Mbvwinji M, Molyneux ME, et al. Bacteraemia and mortality among adult medical admissions in MalawiCpredominance of non-typhi salmonellae and Streptococcus pneumoniae. J Infect 2001;42:44-9.
11. Estrada y Martin RM, Samayoa B, Arathoon E, Mayorga R, Hernandez JE. Atypical infection due to Vibrio cholerae in patients infected with human immunodeficiency virus. Clin Infect Dis 1995;21:1516-7.
12. Linn SC. Cholera bacteraemia in a case of typhoid fever. Chin Med J 1932;46:1092-5.
13. Gunn RA, Kimball PP, Pollard RA, Feeley JC, Feldman RA. Bottle feeding as a risk factor for cholera in infants. Lancet 1979;ii:730-2.
14. Majumdar AS, Ghose AC. Protective properties of anticholera antibodies in human colostrum. Infect Immun 1982;36:962-5.
15. Owen RL, Pierce NF, Apple RT, Cray WC. M cell transport of Vibrio cholerae from the intestinal lumen into Peyer's patches: a mechanism for antigen sampling and for microbial transepithelial migration. J Infect Dis 1986;153:1108-18
